# A novel quantitative PCR detects Babesia infection in patients not identified by currently available non-nucleic acid amplification tests

**DOI:** 10.1186/s12866-017-0929-2

**Published:** 2017-01-14

**Authors:** Lavoisier Akoolo, Samantha Schlachter, Rasel Khan, Laura Alter, Albert D. Rojtman, Kristine Gedroic, Purnima Bhanot, Nikhat Parveen

**Affiliations:** 1 Rutgers New Jersey Medical School, Newark, NJ USA; 2 Meridian Health, Jersey Shore University Medical Center, Neptune, NJ USA; 3 The Gedroic Center, Morristown, NJ USA

**Keywords:** Babesiosis detection, *Babesia microti*, Quantitative PCR, Parasitic disease, Tick-borne infection, Blood-borne disease, Nucleic acid amplification test

## Abstract

**Background:**

Ticks transmit *Babesia microti*, the causative agents of babesiosis in North America and Europe. Babesiosis is now endemic in Northeastern USA and affects people of all ages. *Babesia* species infect erythrocytes and can be transmitted through blood transfusion. Whole blood and blood products, which are not tested for *Babesia,* can cause transfusion-transmitted babesiosis (TTB) resulting in severe consequences in the immuno-compromised patients. The purpose of this study was epidemiological evaluation of babesiosis in a tick-infested state.

**Results:**

We examined blood samples from 192 patients who visited clinics during the active tick-borne diseases season, using a newly developed qPCR assay that uses the specific molecular beacon probe. Due to the absence of clear symptomology, clinical laboratories did not test 131 samples by IFA, FISH or microscopic examination of Giemsa-stained blood smears. *Babesia* infection was detected in all age groups by FISH and microscopy; notably patients >40 years of age represented 64% of tested samples and 13% were younger patients. We tested all samples using qPCR and found that 38% were positive for *Babesia*. Of 28 samples that were positive by FISH, 27 (96%) were also positive by qPCR indicating high congruency between nucleic acid based tests. Interestingly, of 78 asymptomatic samples not tested by FISH, 22 were positive by our qPCR. Direct detection of *Babesia* relies upon microscopic examination of patient blood smears, which is labor intensive, difficult to scale up, requires specific expertise and is hence, often not performed. In fact, a clinical laboratory examined only 23 of 86 blood samples obtained from two different counties by microscopy. By considering individuals positive for *Babesia* infection when results from currently available microscopy, FISH or serological tests were positive, we found that our qPCR is highly sensitive (96.2%) and showed a specificity of 70.5% for *Babesia*.

**Conclusion:**

Robust qPCR using specific probes can be highly useful for efficient and appropriate diagnosis of babesiosis in patients in conjunction with conventional diagnostics, or as a stand-alone test, especially for donated blood screening. The use of a nucleic acid amplification test based screening of blood and blood products could prevent TTB.

**Electronic supplementary material:**

The online version of this article (doi:10.1186/s12866-017-0929-2) contains supplementary material, which is available to authorized users.

## Background

Tick-borne diseases have become increasingly prominent among populations in North American continent and Europe. In fact, Lyme disease caused by *Borrelia burgdorferi* sensu lato infection is the most prevalent arthropod-borne disease in the United States and Europe; however, ticks in both continents are increasingly infected by multiple pathogens [1–6]. A recent report suggests that transmission of *Babesia* species, is promoted by coinfections of reservoir host, the white-footed mouse *Peromyscus leucopus*, with *B. burgdorferi* [7].

Babesiosis case was first reported in 1893 [8]. *B. microti* transmitted by *Ixodes scapularis* ticks is the main cause of human babesiosis in the United States although some cases of *B. duncani* infection have been reported from the Western, coastal states [9]. Babesiosis was first detected in New Jersey in 1969 [10] and has now spread to wider geographic regions, such that in 2011, CDC declared it as a notifiable disease. According to the CDC, *B. microti* infection affects the Northeastern United States - Connecticut, Massachusetts, New Jersey, New York, and Rhode Island along with the Midwestern State of Wisconsin and Minnesota with1636 out of 1744 cases. Together they represented 94% of babesiosis cases in the USA in 2014.

*Babesia* species infects red blood cells and disease can also be transmitted through transfusion of blood affecting already ill recipients. Whole blood and blood products, which are not tested for the presence of *Babesia,* can cause blood transfusion transmitted babesiosis (TTB); resulting in severe consequences in the immuno-compromised patient populations. Due to increasing reported incidence of TTB [11], FDA has recently recommended that blood donors should be screened for *Babesia* infection (http://www.fda.gov/downloads/advisorycommittees/committeesmeetingmaterials/bloodvaccinesandotherbiologics/bloodproductsadvisorycommittee/ucm446274.pdf). From a clinical perspective, infection of humans by *Babesi*a species shows wide-ranging manifestation from asymptomatic to mild flu-like symptoms in immunocompetent people to acute or sometimes fatal disease in splenectomized and immunocompromised individuals or elderly people [11–20]. Patients are often tested for babesiosis only after they manifest hemolytic anemia. *Babesia* species can also transmit through transplacental route [21–23] and can cause jaundice, anemia and neutropenia in infants.

Microscopic examination of Giemsa-stained blood smears provides compelling evidence of infection by *Babesia* species. This method is labor intensive and time-consuming and requires a specific expertise for making correct diagnosis because pleomorphic and non-synchronous trophozoites and ring forms can sometimes make it difficult to identify *Babesia* infection [15]. Recent studies have indicated that Nucleic Acid Testing (NAT) has more promise for diagnosis of babesiosis including among blood donors [24, 25]. Since it is challenging to detect parasitized erythrocytes in early or chronic stages of infection when parasitemia is low or intermittent, serological assays are often found to be more sensitive in detecting infection with *Babesia* species [26–32]; however, these tests cannot detect acute disease before the adaptive immune response is triggered and are also unable to distinguish active disease from the past infections, thus creating a major diagnostic problem particularly in the endemic regions [33, 34]. In this study, we used our novel qPCR test based upon molecular beacon probe with patient samples to evaluate its potential for early detection of babesiosis to prevent further transmission of this protozoan. Thus, we present here detection of Babesia presence in 192 patients from New Jersey by our qPCR assay and compared it with IFA, FISH and microscopy results whenever available.

## Methods

### Samples collection and experimental ethics

Patients presenting different clinical symptoms arrived at Gedroic Center in Morris County, and Meridian hospitals in Ocean and Monmouth Counties of New Jersey and were recommended to get testing for tick borne diseases, either for initial evaluation or follow-up care. At the Gedroic Center, a history of tick bite, and patients presenting with high fever (>102°F) eight weeks after noticing a tick-bite were suspected of suffering from babesiosis. Furthermore, if a patient reported a history of erythema migrans indicating tick-borne infection, or exhibited two of the three symptoms, night sweats, shortness of breath and frontal headaches, a high index of *Babesia* infection was considered and samples were sent to IGeneX for testing. Blood samples were collected from 106 patients who came to the Gedroic Center that also included patients who were considered asymptomatic for babesiosis.

Patient identification criteria for testing at Meridian hospitals/Jersey Shore University Medical Center (JSUMC) included a history of exposure to a tick bite. Patients who did not recall of a tick bite and had some of the following symptoms: fever, plus or minus rash; malaise, fatigue, joint pain; anemia, with or without neutrophilia, and decrease platelet counts were also considered for further testing. Blood testing was conducted via microscopic examination of Giemsa-stained smears at JSUMC.

### Testing of Patient samples by clinical laboratories

Samples collected by Gedroic Center from Morris County, and at JSUMC from Ocean and Monmouth Counties of New Jersey were serially numbered with initials KG and J, respectively. Aliquots of samples collected at Gedroic Center were sent to IGeneX reference laboratory for testing by Fluorescence in situ hybridization (FISH) in which a proprietary fluorescent labeled specific probe is used to directly target the 18S rRNA of *Babesia* species on air dried blood smear on slides. Samples collected in Ocean and Monmouth Counties were sent for testing to core laboratory at JSUMC. Giemsa-stained thin blood smears were examined microscopically for the presence of *Babesia* by the Hematology department of JSUMC and positive smears confirmed by an attending hematologist.

### IFA conducted at NJMS

IFA was conducted for all J samples with four KG samples included as controls. Slides of *Babesia* sp. obtained from Focus Diagnostics were blocked with Phosphate Buffered Saline (PBS) containing 5% bovine serum albumin (BSA) and 5% goat serum for 30 min at room temperature and then probed with 1:128 dilutions of the plasma samples at 37°C for 1h. After three washings with PBS at 5 min intervals, slides were incubated with a mixture of 1:100 dilution of 4′, 6-diamidino-2-phenylindole (DAPI) to stain parasite DNA observed by blue fluorescence and anti-human Alexa 488 conjugated goat antibodies (Molecular Probes, MA). After 1h incubation at 37°C in dark, slides were washed three times with PBS at 5 min interval. Cover glasses were then mounted using 1:1 mixture of glycerol and PBS and sealed with nail enamel to prevent evaporation and drying. Slides were examined by fluorescent microscope using oil emersion objective. Green fluorescence indicated that patient plasma contains antibodies against *B. microti*.

### Culture of *B. microti* and DNA isolation for qPCR

We modified the protocol described previously for *in vitro* cultivation of other *Babesia* species [35] to culture *B. microti*. Briefly, human red blood cells (RBCs) (Valley Biomedical Products, VA) were washed twice with equal volume of RPMI 1640 by mixing and centrifugation at 1,000 rpm on bench top centrifuge. In a T75 flask, a mixture of 13 ml RPMI, 4 ml Fetal Bovine Serum (FBS), 4.5 ml RBCs and ~0.36 mg/ml Gentamicin was inoculated with 0.2 ml of *B. microti* Ingram strain culture stock (ATCC PRA-398), a human babesiosis isolate. The culture was maintained at 37°C in 5% CO_2_ incubator for a few passages only. Giemsa-stained thin smears of culture were observed and parasitemia determined every 2^nd^ day of culture and approximately half of the culture medium from the top of the flask was replaced with fresh medium and RBCs. To determine the sensitivity of detection by qPCR, total DNA was isolated to prepare standard curve. Genomic DNA was isolated from pooled *B. microti* culture from several passages when parasitemia was >2%. Briefly, the infected RBC pellet was washed and then lysed with 2 ml of 0.15% Saponin (Sigma-Aldrich, MO) prepared in PBS by incubation on ice for 30 min. The treated RBCs were centrifuged at 2900 xg at 4°C for 25 min to recover parasite pellet. DNA was isolated following previously described protocol for genomic DNA [36] and concentation determined. For qPCR, 5μl of each dilution was used to prepare the standard curve.

### *B. microti* quantitation by qPCR and Patient samples analyses

Monoplex qPCR was conducted using different dilutions of genomic DNA isolated from *in vitro* grown *B. microti* using the primers and molecular beacon probe for *B. microti* gene encoding Thiamine pyrophosphokinase (*Bmtpk*) as we described previously [36]. Briefly, amplification was performed in 25 μl reaction mixtures containing AmpliTaq Gold PCR reaction buffer (Life Technologies, NY) supplemented with 3 mM MgCl2, 500 ng/μl of bovine serum albumin, 250 μM of each deoxynucleoside triphosphate (dNTP), 500 nM of each primer, 5 units of AmpliTaq Gold polymerase (Life Technologies, NY), and 100 nM each of *Bmtpk* molecular beacon probe. The amplification program consisted of initial heating at 95°C for 10 min, followed by 50 cycles of heating at 95°C for 15s, annealing and fluorescence detection at 60°C for 30s, and polymerization at 72°C for 20s. Based upon the genome size (6.5 Mb), of *B. microti* [37], 8ng of DNA was calculated to contain 10^6^ copies of *Bmtpk* gene. A standard curve was prepared from this assay using 5-fold dilutions of *B. microti* genomic DNA (Fig. 2b). DNA from blood was isolated using the method described previously in details [36]. Duplex assays using the conditions as described above were conducted with patient whole blood samples that also contain leukocytes such that human gene as internal control could be incorporated in the tests. Primers and molecular beacon probe for human *actA1* gene were included as internal control for patient samples in the duplex assays, to ensure the quality of patient blood DNA was suitable for PCR [36]. QPCR for each patient sample was conducted at least three times to confirm results, especially when results from alternative tests were not available.

#### Statistical analyses

Statistical analyses were conducted ﻿by Rutgers University Biostatistics and E﻿pidemiology Services Cente﻿r﻿.﻿ Patients were defined as positive for *Babesia* infection when IFA/FISH and/or microscopy results were positive. Otherwise the babesiosis was considered negative for comparison with qPCR results. We determined the sensitivity of qPCR as a measure of the proportion of subjects that tested positive as compared to the disease state of the subject based upon clinicians’ determination and accordingly tests were conducted in this study, i.e., *Babesia* FISH, IFA and microscopy. Specificity measure in our study indicated samples negative by qPCR that were truly negative for babesiosis by criteria described above. The positive predictive value (PPV) determination indicates that the proportion of subjects that will be considered positive for *Babesia* infection given that they tested positive by qPCR, and the negative predictive value (NPV) determined the proportion of subjects that will not have the disease if they tested negative by qPCR. No previous report based upon qPCR based patient samples have determined PPV and NPV of their assay probably due to the lack of a gold standard assay available for babesiosis.

## Results

### Study population and babesiosis diagnosis

**Fig. 1:**
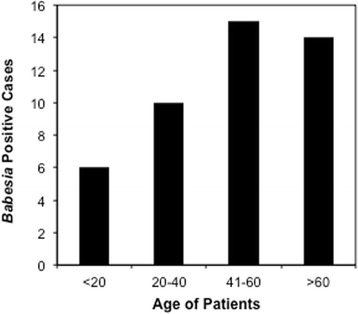
*B. microti* infection relationship with the age of patients. Whole blood samples obtained from 192 patients from Morris, Ocean and Monmouth Counties of New Jersey examined by FISH or microscopic examination of Giemsa-stained thin smears indicates a higher babesiosis incidence in patients older than 40 years of age

We examined 192 blood samples collected in 2015 in Ocean, Monmouth and Morris Counties from patients ranging in age from 2 to 90 years. Among 106 samples from Gedroic Center, 78 were asymptomatic and 28 symptomatic. Sixty three samples from JSUMC were considered asymptomatic and 23 symptomatic. Only blood sample smears from symptomatic patients stained with Giemsa stain were examined microscopically (Table 1). Thus, FISH was only conducted for 37/106 samples from the Gedroic Center and none of 86 samples from JSUMC. IFA at NJMS was conducted primarily with JSUMC samples with four samples from Gedroic Center, 3 FISH negative and 1 FISH positive included as controls. Only 23 blood samples were examined by microscopy at JSUMC and 4 from Gedroic Center by CDC. *Babesia* was detected in three patients by routine peripheral smear evaluation without suspicion of a tick bite infection in their differential diagnosis. Ninety-five patients or approximately 50% of the patient sample pool represented individuals older than 40 years (Fig. 1). Forty five (23%) patient samples from three counties were positive for infection by either blood smear analysis or FISH. All age groups were affected with 64% of the babesiosis positive cases older than 40 years (Fig. 1); notably, 13% of younger cases were positive.

**Table 1:** Analysis of qPCR, conducted in a blinded manner to detect the presence of *B. microti* DNA and comparison with *Babesia* FISH, IFA and microscopic examination of Giemsa-stained thin blood smear

qPCR Results from 3 repeats (No. of Samples)#	IFA			Babesia FISH			Microscopy of Giemsa-Stained blood smear		
	Positive	Negative	NT	Positive	Negative	NT	Positive/±	Negative	NT
+/+/+ (3)	0	0	3	2	0	1	1	0	2
+/+/- (65)	7/1±	19	38	7	5	53	8/1 ± ^a^	4	52
+/-/- (24)	0	2	22	9	0	15	7	0	17
-/-/- (100)	1	40	59	1	13	86	0	7	93

#Each plus or minus sign indicates the results from one qPCR assay. Patient samples were considered positive or negative by qPCR based upon comparison with the standard curve in Fig. 2b

^a^Microscopic examination of Giemsa-stained blood smears provided uncertain results for *Babesia* presence. IFA ± indicates only some of the parasites showed fluorescence

NT-Not Tested. NT samples were considered negative for diagnosis of babesiosis by physicians based upon symptomatology and also for statistical analyses in this study

### Detection of *B. microti* by qPCR

**Fig. 2:**
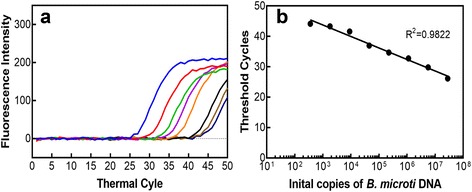
Determine the sensitivity of detection of *B. microti* using *in vitro* grown parasites by qPCR. **a** Amplification plots of *Bmtpk* gene in monoplex qPCR assay starting with 10^6^ gene copies (8ng DNA). Five-fold dilutions of genomic DNA of *B. microti* purified from *in vitro* grown culture using *Bmtpk* primers and molecular beacon probe were used to determine quantities of *B. microti*. Dotted line indicates ‘no template’ control. **b** A high coefficient of correlation (*r* ^2^ = 0.9822) between the amplification cycle number (Ct values) and *Bmtpk* copy number representing the parasite numbers obtained from the standard curve indicates that qPCR can be used effectively to evaluate even low level of parasitemia in patients

We compared our qPCR data with the results of samples tested in the clinical laboratories either by microscopy (Monmouth and Ocean Counties) or those obtained from IGeneX using *Babesia* FISH (Morris County) and also evaluated untested samples. In our qPCR assays, different DNA dilutions (Fig. 2a) were used to prepare a standard curve (Fig. 2b). We conducted monoplex qPCR assay because erythrocytes rather than whole blood is used for *in vitro* culture of *B. microti*. Based upon the standard curve and repeat of experiment, we estimated that consistent sensitivity of detection of our qPCR assay is approximately 360 copies of the *B. microti* Thiamine pyrophosphokinase (*Bmtpk*) amplicon.

### Concordance of positive babesiosis results with qPCR, microscopy and IFA testing

Duplex qPCR results in our laboratory with patient samples that included amplification and detection of gene encoding Actin A1 as internal control as compared with the monoplex assay using *in vitro* culture of *B. microti* indicated that parasitemia was often low in patients. We previously demonstrated that the use of molecular beacon probes provides low background, and sensitivity of detection of *Babesia* spp., is not affected in our duplex and even multiplex assays as compared to monoplex test [36]. Detection of *B. microti* in samples using our qPCR demonstrated high correlation with FISH such that of 28 samples positive by FISH, 27 (96%) were positive by qPCR. There was less concordance between FISH negative and qPCR results such that 72% (13/18) of FISH negative samples were also negative by qPCR (Additional file 1: Table S1). Due to blood samples storage for a week at 4°C prior to making smears, blood smears were difficult to confirm for *Babesia* presence at NJMS. Therefore, three blood sample (from Gedroic Center) smears that looked potentially positive and one clearly negative were sent to CDC for evaluation. Smear quality affected confirmatory determination at CDC too and were considered negative for this analysis.

**Fig. 3:**
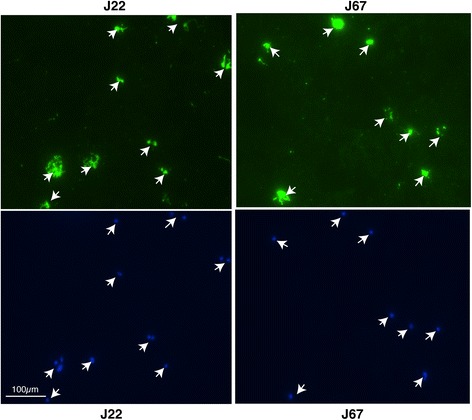
Detection of *B. microti* presence in the qPCR positive patient samples by IFA (*Top Panels*) Two representative samples (J22 and J67) from Ocean and Monmouth Counties not tested for *Babesia* by microscopy at JSUMC show green fluorescence due to reactivity of antibodies in patient plasma with the parasites followed by detection with Alexa 488 conjugated secondary antibodies, when observed by using FITC filter indicating positive IFA results (marked by arrows). (*Bottom Panels*) Blue fluorescence due to DAPI staining shows the parasites present (marked by arrows) in each field of view of the Nikon 80i fluorescence microscope at × 1000 magnification when Apo-Plan TIRF objective was used. Scales shown represent respective panels of Giemsa-stained microscopy and IFA

We used qPCR results for patient samples as positive if the cycle number could be plotted on the standard curve in Fig. 2. Our qPCR results also showed significant (96%) agreement with microscopy data obtained at JSUMC such that of seven samples confirmed negative by microscopy, only one was positive by qPCR (Table 1, and Additional file 1: Table S1). Seven of sixteen microscopically positive samples (at JSUMC) tested positive by qPCR only once, most likely due to stochastic detection in patient blood samples with low parasitemia. Of the sixty-two samples that were not tested for *Babesia* at JSUMC, twenty-four samples (39%) were positive by qPCR at least twice. Among these twenty-four samples, eight were also positive by IFA (Table 1, Additional file 1: Table S1 and Fig. 3). Of 29 qPCR positive samples tested by IFA, 21 were IFA negative and looked similar to the negative controls (Additional file 1: Table S1), suggesting that plasma of these patients did not yet possess antibodies against *Babesia* antigens. In any case, we were able to verify our qPCR results for some samples by IFA.

A practical “Gold standard” test is not available for detection of *Babesia* infection at present, and highlights the need for better methods of detection. In this study, we defined babesiosis based-upon the results of the microscopy, FISH and/or IFA such that a positive result for each of these tests was considered a positive disease state, otherwise it was considered as negative for babesiosis (Table 2). We also summarized results presented in Table 1 for statistical analysis in Table 3. Based on these assignments, there were 53 positive disease states and 139 negative babesiosis cases among 192 patients.

**Table 2:** Defining babesiosis disease state based upon *Babesia* FISH, IFA and microscopic examination of Giemsa–stained patient blood smears

		*Giemsa-staining and Microscopy*			
		Positive	Negative	Not tested/Indefinite	Total
*FISH/IFA*	Positive	0	1	36	37^a^ (19.3%)
	Negative	0	7	128	135 (70.3%)
	Not tested	16	4	0	20 (10.4%)
	Total	16^a^ (8.3%)	12 (6.3%)	164 (85.4%)	192

^a^Indicates the assignment of positive babesiosis state

**Table 3:** Summary table of qPCR results comparison with the serological tests and microscopy used for statistical analyses, and PPV and NPV determinations

	Babesiosis Disease Present	Babesiosis Disease Absent	Total
Positive qPCR	51	41	92 (47.9%)
Negative qPCR	2	98	100 (52.1%)
Total	53 (27.6%)	139 (72.4%)	192

### Data Analyses from Table 3

$$ Sensitivity = \frac{\#\ \mathrm{of}\ \mathrm{True}\ \mathrm{Positives}}{\#\ \mathrm{with}\ \mathrm{Disease}} = \frac{51\ }{53} = 0.962=96.2\% $$

$$ Standard\  Error\  of\  Sensitivity = \sqrt{\frac{Sensitivity*\left(1- Sensitivity\right)}{\#\ \mathrm{with}\ \mathrm{Disease}}} = \sqrt{\frac{0.962*\left(1-0.962\right)}{53}}=0.026=2.6\% $$

$$ Specificity = \frac{\#\ \mathrm{of}\ \mathrm{True}\ \mathrm{Negatives}}{\#\ \mathrm{without}\ \mathrm{Disease}} = \frac{98\ }{139} = 0.705=70.5\% $$

$$ Standard\  Error\  of\  Specificity = \sqrt{\frac{Specificity*\left(1- Specificity\right)}{\#\ \mathrm{without}\ \mathrm{Disease}}} = \sqrt{\frac{0.705*\left(1-0.705\right)}{139}}=0.039=3.9\% $$

$$ PPV = \frac{\#\ \mathrm{of}\ \mathrm{True}\ \mathrm{Positive}\mathrm{s}}{\#\ \mathrm{of}\ \mathrm{Positive}\ \mathrm{Calls}} = \frac{51\ }{92} = 0.554=55.4\% $$

$$ Standard\  Error\  of\ PPV = \sqrt{\frac{PPV*\left(1-PPV\right)}{\#\ \mathrm{of}\ \mathrm{Positive}\ \mathrm{Calls}}} = \sqrt{\frac{0.554*\left(1-0.554\right)}{92}}=0.052=5.2\% $$

$$ NPV = \frac{\#\ \mathrm{of}\ \mathrm{True}\ \mathrm{Negative}\mathrm{s}}{\#\ \mathrm{of}\ \mathrm{Negative}\ \mathrm{Calls}} = \frac{98\ }{100} = 0.980=98.0\% $$

$$ Standard\  Error\  of\ NPV = \sqrt{\frac{NPV*\left(1-NPV\right)}{\#\ \mathrm{of}\ \mathrm{Negative}\ \mathrm{Calls}}} = \sqrt{\frac{0.98*\left(1-0.98\right)}{100}}=0.014=1.4\% $$

All 95% confidence intervals were calculated as:

$$ Confidence\  Interval= Point\  Estimate \pm 1.96* Standard\  Error $$

By analysis of the qPCR results as compared with the babesiosis disease state as calculated above, the sensitivity of qPCR was determined to be 96.2% with a 95% confidence interval of 91.1 to 100% (Table 4). The specificity of qPCR was 70.5% with a 95% confidence interval of 62.9 to 78.1%. The PPV of qPCR was 55.4% (51/92) with a 95% confidence interval of 45.3–65.6% and the NPV was found to be 98.0% (98/100) with a 95% confidence interval of 95.3–100% (Table 4).

**Table 4:** Metrics of qPCR efficacy compared to the positive or negative babesiosis disease state as determined by IFA and microscopy

qPCR Metrics	Point Estimate	95% Confidence Interval (Lower Bound, Upper Bound)*	Standard Error
Sensitivity	96.2%	(91.1%, 100.0%)	2.6%
Specificity	70.5%	(62.9%, 78.1%)	3.9%
Positive Predictive Value	55.4%	(45.3%, 65.6%)	5.2%
Negative Predictive Value	98.0%	(95.3%, 100.0%)	1.4%

*Confidence intervals with upper bounds >100% are shown as 100%

## Discussion

We report here testing for *Babesia* spp. in 192 patient blood samples using our qPCR assay [36] and compared the results with those obtained by microscopic examination of Giemsa-stained blood smears, *Babesia* FISH or by serology. A significant association of babesiosis with patients’ older age (>40 years) in New Jersey here is similar to the pattern reported by CDC based upon 1,762 babesiosis cases from twenty seven states in the USA in 2013. A higher percentage of younger patients, 13% in our sample set versus approximately 3% cases by CDC, indicated that in New Jersey babesiosis affects a broader age range of individuals and is not just a phenomenon of older age. Thus, babesiosis is a public health concern for all populations.

Groups of patients who were asymptomatic, exhibited non-specific symptoms, or lacked spectrum of symptoms considered by physicians to suspect *Babesia* infection were not tested by clinical laboratories, such that only 12% of patient samples were examined microscopically in this study. Recently, PCR/qPCR assays have been developed by some laboratories to detect the presence of *Babesia* species [25, 34, 36, 38–41]. These tests were found to be more sensitive than microscopic examination particularly when parasitemia is low. In our study, we found 96% agreement between our qPCR test results with those obtained by microscopic examination at JSUMC or with FISH, demonstrating the accuracy of the qPCR assay. Furthermore, 100% concordance of our qPCR among microscopically positive samples (Table 1) demonstrates the accuracy of our qPCR. Even though qPCR provides a better approximation of parasitemia and will be very useful before treatment, clinicians should consider the caveat that DNA of pathogens often persists at least for a limited time after completion of treatment regimen as was recently reported using a qPCR method involving 18S rRNA gene amplicon and respective primers and probe [41]. Ultimately nucleic acids are degraded by nucleases present in plasma. In addition, twenty-four of sixty-two (39%) samples not tested for babesiosis at the clinic also showed positive results with our qPCR assay, validating the importance of this method as a diagnostic assay for field application. These results further emphasize the success of our test in detection of *Babesia* infection in patients at different stages of infection.

In total, 71% qPCR positive samples from all three counties were positive for babesiosis by IFA. Serological tests are indirect tests that measure the host response to an infection and are not reliable indicators of active infection. High sensitivity of qPCR (96.4%) indicates that serological tests or microscopic examination of smears after Giemsa staining and FISH are useful for positive outcome before treatment; however, negative results by these tests do not necessarily indicate the absence of infection by *Babesia* species. In the absence of an efficient gold standard diagnostic assay, negative serological results during early stage of disease or by negative microscopic observation due to low parasitemia could be responsible for lower specificity (70.5%) of our qPCR for babesiosis. To verify our results, qPCR was conducted at least three times for each sample (Table 1). Data from at least one alternative test was generated for all patient samples. Samples that tested positive only once by qPCR likely indicate stochastic detection of *Babesia* when parasitemia is low. To support these qPCR results, we also tested samples by IFA. In fact, among16 samples positive once by qPCR, 9 were also found to be positive on microscopic examination, validating our qPCR assay. Detection of *Babesia* by qPCR in 22/78 negative/untested samples (28%) at Gedroic Center may represent acute disease. However, we cannot rule out the possibility of false-positive result when qPCR is positive only 1/3 times it is tested and when results from an alternative test are not available. A high NPV (98.0%) determination for qPCR indicates that the negative qPCR results have a very high level of agreement with a negative disease. Relatively lower PPV (58.7%) could be attributed to detection of the early babesiosis in patients by our sensitive molecular beacon probe-based qPCR assay when parasitemia is very low making microscopy difficult and because adaptive immune response has not yet established in patients. Thus, positive qPCR individuals do not show positive disease state by FISH/IFA tests or by microscopic examination of Giemsa-stained blood smears. Supporting this premise, patient samples in our study often showed parasitemia at the lower end of detection by qPCR as determined by comparison with data obtained from *in vitro* grown *B. microti* (Fig. 2).

Overall, we found that our qPCR assay is more sensitive, reliable and fast method for detection of *Babesia* infection. Our qPCR results together with those from complementary tests presented here suggest that incidence of babesiosis has increased significantly from less than 50 reported cases in New Jersey state in 2001 to 92 case in 2015 just in 3/21 NJ counties. This could be partially attributed to increased awareness and testing of patients for babesiosis. Since our samples may be a representation of the pattern observed in other endemic regions, testing of patient samples in triplicate by qPCR with at least two positive results will provide rapid, efficient and sensitive diagnostic of babesiosis and will facilitate design of proper treatment regimens. This method could also be widely implemented for screening of blood donors, and for testing donated blood and blood components in order to reduce the risk of TTB.

## Conclusions

Results of our novel qPCR assay with patient blood samples for babesiosis were compared with those obtained by FISH, IFA and microscopy. A combined positive rate of 47% indicates a high occurrence of babesiosis in three counties of New Jersey. Furthermore, this study confirms that currently used tests do not reliably detect early acute babesiosis especially when parasitemia is low and patients are asymptomatic. Diagnostic tests, such as time consuming and labor intensive microscopic examination of thin blood smears or *Babesia* FISH, are often not carried out when disease symptoms are not clear. These tests are particularly not feasible for large scale screening purposes and cannot be automated. Therefore, the currently used tests together result in underestimation of the incidence of babesiosis, as has also been reported for Lyme disease in the endemic regions of tick-borne diseases. Our qPCR assay using molecular-beacon probes is highly sensitive, and showed significant specificity, PPV and NPV for detection of *B. microti* presence in patients’ blood and could become an efficient stand-alone diagnostic test, particularly for blood screening, or when combined with IFA and/or microscopy.

## Additional file

Additional file 1: A novel quantitative PCR detects Babesia infection in patients not identified by currently available non-nucleic acid amplification tests. **Table S1**. Analyses of patient blood samples tested by qPCR, FISH, IFA and microscopic examination of Giemsa-stained blood smears. (DOCX 17 kb)

## Abbreviations

JSUMC: Jersey Shore University Medical Center
NPV: Negative predictive value
PPV: Positive predictive value
qPCR: Real-time quantitative PCR
RBCs: Red blood cells
TTB: Transfusion transmitted babesiosis

## References

[1] Skotarczak B, Rymaszewska A, Wodecka B, Sawczuk M (2003). Molecular evidence of coinfection of Borrelia burgdorferi sensu lato, human granulocytic ehrlichiosis agent, and Babesia microti in ticks from northwestern Poland. J Parasitol.

[2] Franke J, Fritzsch J, Tomaso H, Straube E, Dorn W, Hildebrandt A (2010). Coexistence of pathogens in host-seeking and feeding ticks within a single natural habitat in Central Germany. Appl Environ Microbiol.

[3] Aliota MT, Dupuis AP, Wilczek MP, Peters RJ, Ostfeld RS, Kramer LD (2014). The prevalence of zoonotic tick-borne pathogens in Ixodes scapularis collected in the Hudson Valley, New York State. Vector Borne Zoonotic Dis.

[4] Lommano E, Bertaiola L, Dupasquier C, Gern L (2012). Infections and coinfections of questing Ixodes ricinus ticks by emerging zoonotic pathogens in Western Switzerland. Appl Environ Microbiol.

[5] Hersh MH, Ostfeld RS, McHenry DJ, Tibbetts M, Brunner JL, Killilea ME, LoGiudice K, Schmidt KA, Keesing F (2014). Co-infection of blacklegged ticks with Babesia microti and Borrelia burgdorferi is higher than expected and acquired from small mammal hosts. PLoS One.

[6] Eshoo MW, Crowder CD, Carolan HE, Rounds MA, Ecker DJ, Haag H, Mothes B, Nolte O (2014). Broad-range survey of tick-borne pathogens in Southern Germany reveals a high prevalence of Babesia microti and a diversity of other tick-borne pathogens. Vector Borne Zoonotic Dis.

[7] Dunn JM, Krause PJ, Davis S, Vannier EG, Fitzpatrick MC, Rollend L, Belperron AA, States SL, Stacey A, Bockenstedt LK (2014). Borrelia burgdorferi promotes the establishment of Babesia microti in the northeastern United States. PLoS One.

[8] Ouhelli H, Schein E (1988). Effect of temperature on transovarial transmission of Babesia bigemina (Smith and Kilborne, 1893) in Boophilus annulatus (Say, 1821). Vet Parasitol.

[9] Centers for Disease C, Prevention (2012). Babesiosis surveillance - 18 States, 2011. MMWR Morb Mortal Wkly Rep.

[10] Western KA, Benson GD, Gleason NN, Healy GR, Schultz MG (1970). Babesiosis in a Massachusetts resident. N Engl J Med.

[11] Herwaldt BL, Linden JV, Bosserman E, Young C, Olkowska D, Wilson M (2011). Transfusion-associated babesiosis in the United States: a description of cases. Ann Intern Med.

[12] Hunfeld KP, Hildebrandt A, Gray JS (2008). Babesiosis: recent insights into an ancient disease. Int J Parasitol.

[13] Kjemtrup AM, Conrad PA (2000). Human babesiosis: an emerging tick-borne disease. Int J Parasitol.

[14] Herman JH, Ayache S, Olkowska D (2010). Autoimmunity in transfusion babesiosis: a spectrum of clinical presentations. J Clin Apher.

[15] Setty S, Khalil Z, Schori P, Azar M, Ferrieri P (2003). Babesiosis. Two atypical cases from Minnesota and a review. Am J Clin Pathol.

[16] Sinski E, Welc-Faleciak R, Poglod R (2011). Babesia spp. infections transmitted through blood transfusion. Wiad Parazytol.

[17] Chiang E, Haller N (2011). Babesiosis: an emerging infectious disease that can affect those who travel to the northeastern United States. Travel Med Infect Dis.

[18] Holler JG, Roser D, Nielsen HV, Eickhardt S, Chen M, Lester A, Bang D, Frandsen C, David KP (2013). A case of human babesiosis in Denmark. Travel Med Infect Dis.

[19] Poisnel E, Ebbo M, Berda-Haddad Y, Faucher B, Bernit E, Carcy B, Piarroux R, Harle JR, Schleinitz N (2013). Babesia microti: an unusual travel-related disease. BMC Infect Dis.

[20] van Vugt M, Wetsteyn JC, Haverkort M, Kolader M, Verhaar N, Spanjaard L, Grobusch MP, Bart A, van Gool T (2011). New England souvenirs. J Travel Med.

[21] Luckett R, Rodriguez W, Katz D (2014). Babesiosis in pregnancy. Obstet Gynecol.

[22] Joseph JT, Roy SS, Shams N, Visintainer P, Nadelman RB, Hosur S, Nelson J, Wormser GP (2011). Babesiosis in lower hudson valley, new york, USA. Emerg Infect Dis.

[23] Joseph JT, Purtill K, Wong SJ, Munoz J, Teal A, Madison-Antenucci S, Horowitz HW, Aguero-Rosenfeld ME, Moore JM, Abramowsky C (2012). Vertical transmission of *Babesia microti*, United States. Emerg Infect Dis.

[24] Cushing M, Shaz B (2012). Transfusion-transmitted babesiosis: achieving successful mitigation while balancing cost and donor loss. Transfusion.

[25] Jahfari S, Hofhuis A, Fonville M, van der Giessen J, van Pelt W, Sprong H (2016). Molecular Detection of Tick-Borne Pathogens in Humans with Tick Bites and Erythema Migrans, in the Netherlands. PLoS Negl Trop Dis.

[26] Magnarelli LA JWIJ, Dumler JS, Heimer R, Fikrig E (1998). Reactivity of human sera to different strains of granulocytic ehrlichiae in immunodiagnostic assays. J Infect Dis.

[27] Yoshinari NH, Abrao MG, Bonoldi VL, Soares CO, Madruga CR, Scofield A, Massard CL, da Fonseca AH (2003). Coexistence of antibodies to tick-borne agents of babesiosis and Lyme borreliosis in patients from Cotia county, State of Sao Paulo, Brazil. Mem Inst Oswaldo Cruz.

[28] Foppa IM, Krause PJ, Spielman A, Goethert H, Gern L, Brand B, Telford SR (2002). Entomologic and serologic evidence of zoonotic transmission of Babesia microti, eastern Switzerland. Emerg Infect Dis.

[29] Hunfeld KP, Ernst M, Zachary P, Jaulhac B, Sonneborn HH, Brade V (2002). Development and laboratory evaluation of a new recombinant ELISA for the serodiagnosis of Lyme disease. Wien Klin Wochenschr.

[30] Lempereur L, Shiels B, Heyman P, Moreau E, Saegerman C, Losson B, Malandrin L (2015). A retrospective serological survey on human babesiosis in Belgium. Clin Microbiol Infect.

[31] Mayne PJ (2015). Clinical determinants of Lyme borreliosis, babesiosis, bartonellosis, anaplasmosis, and ehrlichiosis in an Australian cohort. Int J Gen Med.

[32] Vannier E, Gewurz BE, Krause PJ (2008). Human babesiosis. Infect Dis Clin North Am.

[33] Fox LM, Wingerter S, Ahmed A, Arnold A, Chou J, Rhein L, Levy O (2006). Neonatal babesiosis: case report and review of the literature. Pediatr Infect Dis J.

[34] Wormser GP, Villafuerte P, Nolan SM, Wang G, Lerner RG, Saetre KL, Maria MH, Branda JA (2015). Neutropenia in Congenital and Adult Babesiosis. Am J Clin Pathol.

[35] Guan G, Ma M, Liu A, Du P, Ren Q, Li Y, Wang J, Liu Z, Yin H, Luo J (2012). Continuous in vitro cultivation of a recently identified Babesia that infects small ruminants in China. Vet Parasitol.

[36] Chan K, Marras SA, Parveen N (2013). Sensitive multiplex PCR assay to differentiate Lyme spirochetes and emerging pathogens Anaplasma phagocytophilum and Babesia microti. BMC Microbiol.

[37] Cornillot E, Hadj-Kaddour K, Dassouli A, Noel B, Ranwez V, Vacherie B, Augagneur Y, Bres V, Duclos A, Randazzo S (2012). Sequencing of the smallest Apicomplexan genome from the human pathogen Babesia microti. Nucleic Acids Res.

[38] Teal AE, Habura A, Ennis J, Keithly JS, Madison-Antenucci S (2012). A new real-time PCR assay for improved detection of the parasite *Babesia microti*. J Clin Microbiol.

[39] Hong SH, Anu D, Jeong YI, Abmed D, Cho SH, Lee WJ, Lee SE (2014). Molecular detection and seroprevalence of Babesia microti among stock farmers in Khutul City, Selenge Province, Mongolia. Korean J Parasitol.

[40] Moniuszko A, Dunaj J, Swiecicka I, Zambrowski G, Chmielewska-Badora J, Zukiewicz-Sobczak W, Zajkowska J, Czupryna P, Kondrusik M, Grygorczuk S (2014). Co-infections with Borrelia species, Anaplasma phagocytophilum and Babesia spp. in patients with tick-borne encephalitis. Eur J Clin Microbiol Infect Dis.

[41] Wang G, Wormser GP, Zhuge J, Villafuerte P, Ip D, Zeren C, Fallon JT (2015). Utilization of a real-time PCR assay for diagnosis of Babesia microti infection in clinical practice. Ticks Tick Borne Dis.

