# Supplementary material for: A novel quantitative PCR detects Babesia infection in patients not identified by currently available non-nucleic acid amplification tests
Source: BMC Microbiol. 2017 Jan 14;17:16. doi: 10.1186/s12866-017-0929-2 (PMC5237571; doi:10.1186/s12866-017-0929-2)
Supplement: Additional file 1: — A novel quantitative PCR detects Babesia infection in patients not identified by currently available non-nucleic acid amplification tests. Table S1. Analyses of patient blood samples tested by qPCR, FISH, IFA and microscopic examination of Giemsa-stained blood smears. (DOCX 17 kb) [file 12866_2017_929_MOESM1_ESM.docx]

**Additional file 1**

**Table S1. Analyses of patient blood samples tested by qPCR, FISH, IFA and microscopic examination of Giemsa-stained blood smears.**

| **Samples (Total number of samples)** | **qPCR** | **IFA** | **Babesia FISH** | **Microscopy** |
| --- | --- | --- | --- | --- |
| J1, J2, J17, J18, J19, J20, J31, J35, J40, J51, J53, J56, J57, J78, J86 **(15)** | Positive | NT | NT | Positive |
| J8 **(1)** | Positive | Negative | NT | Positive |
| KG36, KG72, KG103 **(3)** | Positive | NT | Negative | NT |
| J10, J11, J13, J15, J24, J25, J29, J39, J43, J49, **J55,** J72, J74, J80, J81, J82, J85, J87 **(18)** | Positive | 17 Negative, **1±** | NT | NT |
| KG45 **(1)** | Positive | NT | Negative | Negative* |
| KG48, KG49, KG99 **(3)** | Positive | NT | NT | Negative* |
| KG5, KG6, KG7, KG10, KG13, KG16, KG17, KG27, KG53, KG54, KG57, KG60, KG78, KG85, KG93, KG95, KG98, KG100 **(18)** | Positive | NT | Positive | NT |
| J22, J36, J37, J42, J58, J64, J67 **(7)** | Positive | Positive | NT | NT |
| KG1, KG2, KG3, KG8, KG9, KG11, KG12, KG24, KG29, KG41, KG43, KG46, KG56, KG63, KG68, KG75, KG84, KG94, KG96, KG97, KG104, KG105, KG106 **(23)** | Positive | NT | NT | NT |
| **J23,** J41 **(2)** | Positive | Negative | NT | 1 Negative, **1±** |
| KG86 **(1)** | Positive | Negative | Negative | NT |
| J32, J38, J46, J66, J75 **(5)** | Negative | NT | NT | Negative |
| KG18, KG25, J3, J4, J5, J6, J7, J9, J14, J16, J21, J26, J27, J28, J30, J33, J34, J44, J45, J47, J48, J52, J54, J59, J60, J62, J63, J65, J68, J69, J70, J71, J73, J76, J77, J79, J83, J84 **(38)** | Negative | Negative | NT | NT |
| KG42, KG44, KG62, KG64, KG65, KG66, KG70, KG71, KG73, KG74, KG77, KG87 **(12)** | Negative | NT | Negative | NT |
| KG4, KG14, KG15, KG19, KG20, KG21, KG22, KG23, KG26, KG28, KG30, KG31, KG32, KG33, KG34, KG35, KG37, KG38, KG39, KG40, KG47, KG50, KG51, KG52, KG55, KG58, KG59, KG61, KG67, KG76, KG80, KG81, KG82, KG83, KG88, KG89, KG90, KG91, KG92, KG101, KG102 **(41)** | Negative | NT | NT | NT |
| J12 **(1)** | Negative | Positive | NT | NT |
| KG69 **(1)** | Negative | NT | Positive | NT |
| J50 **(1)** | Negative | Negative | NT | Negative |
| KG79 **(1)** | Negative | Negative | Negative | NT |
| **Total tested (192)** | 192 | 70 | 37 | 28 |

NT- Not Tested (because physicians considered negative based upon symptoms). Only samples at Gedroic Center were tested by FISH and JSUMC by microscopic examination of Giemsa-stained blood smears, *microscopic examination of four KG samples was done at CDC. Samples marked in bold in the left column show **±** results**.**
